# Case Series of Intrauterine Subamniotic Hemorrhage

**DOI:** 10.1155/2019/1828457

**Published:** 2019-05-29

**Authors:** Momoko Owada, Yoshie Shibata, Shunji Suzuki

**Affiliations:** Department of Obstetrics and Gynecology, Japanese Red Cross Katsushika Maternity Hospital, Tokyo, Japan

## Abstract

We present here 9 cases of intrauterine subamniotic hemorrhage to describe the pathological sequence. The definite diagnosis of subamniotic hemorrhage was mainly made macroscopically with the presence of hemorrhage or hematoma wrapped in the membrane on the fetal surface by postnatal examining of the placenta. In 7 of them (78%), the presence of gradual subamniotic hemorrhage in the second trimester of pregnancy was suspected. In the cases, severe fetal growth restriction and preterm delivery were recognized in 2 (29%) and 4 cases (57%), respectively. In the other 2 cases (22%), acute anemia associated with acute subamniotic hemorrhage was clinically suspected. This may be the first report examining the clinical characteristics of acute or gradual intrauterine subamniotic hemorrhage. Both patterns of subamniotic hemorrhage seemed to be associated with the adverse perinatal outcomes.

## 1. Introduction

Subamniotic hemorrhage is defined as a hemorrhage between the amniotic membrane and the fetal chorionic plate, following a tear in one of the branches of an umbilical vessel. It is sometimes seen in cases of excessive umbilical cord traction during the third stage of delivery [1, 2] associated with the raised umbilical venous pressure [3]. However, the occurrence of intrauterine subamniotic hemorrhage is relatively rare. Vessel trauma leading to subamniotic hemorrhage in the latter sequence has been supposed to be caused by sudden traction due to fetal movements or to external pressure or spontaneously by the intravascular pressure at a focus of lower vascular wall resistance [3]. To date, some cases concerning various pathological mechanisms associated with subamniotic hemorrhage have been reported [1, 2, 4–8].

We present here our 9 cases of subamniotic hemorrhage to describe the pathological sequence.

## 2. Case Series

The material reviewed consisted of the total population of women who delivered at 22 weeks' gestation or later at Japanese Red Cross Katsushika Maternity Hospital between 2006 and 2015. All the data are stored in a computer of our hospital. In our institute, all placentae were screened identically by trained staff (plural Japanese obstetrics specialists). Informed consent concerning analysis from a retrospective database was obtained from all subjects. The definite diagnosis of subamniotic hemorrhage was made macroscopically with the presence of hemorrhage or hematoma wrapped in the membrane on the fetal surface postnatally and confirmed by placental pathology with hematoma lying between the chorionic plate and amnion associated with the rupture of chorionic vessels.

During the study period, there were 20,118 deliveries at 22 weeks' gestation or later at our hospital. In 9 of them, subamniotic hemorrhage was confirmed in the placenta postnatally as shown in Table 1. Therefore, the incidence of subamniotic hemorrhage that can be confirmed postnatally is estimated to be about 0.04%.

**Figure 1:**
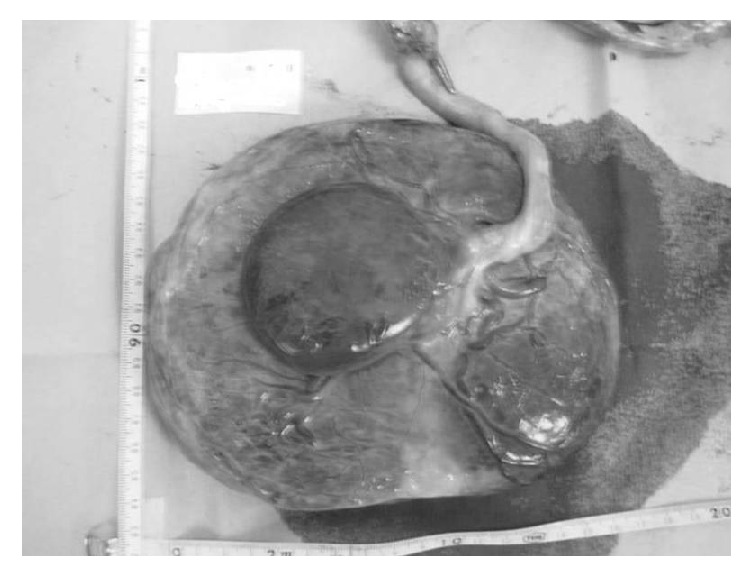
Macroscopic view of the placenta in Case 4 at delivery, showing an oval-shaped structure wrapped in the amniotic membrane.

**Figure 2:**
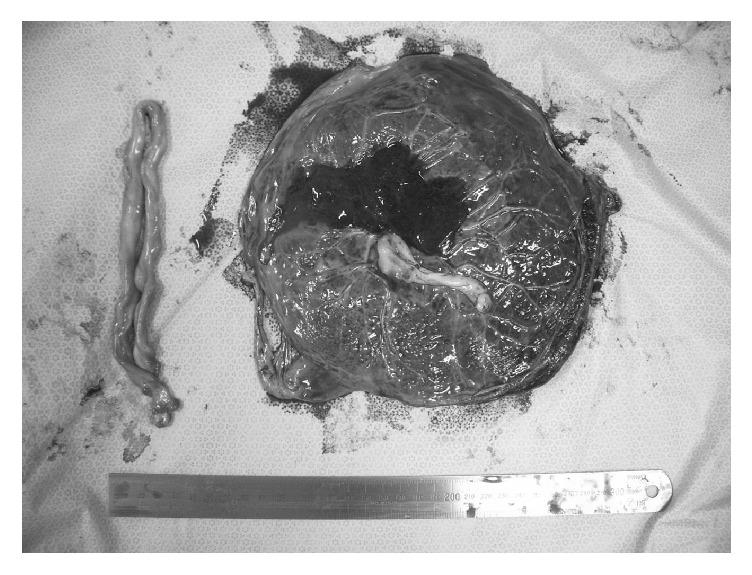
Macroscopic view of the placenta in Case 8 at delivery, showing marginal irregular traces of bleeding under the broken amniotic membrane.

**Figure 3:**
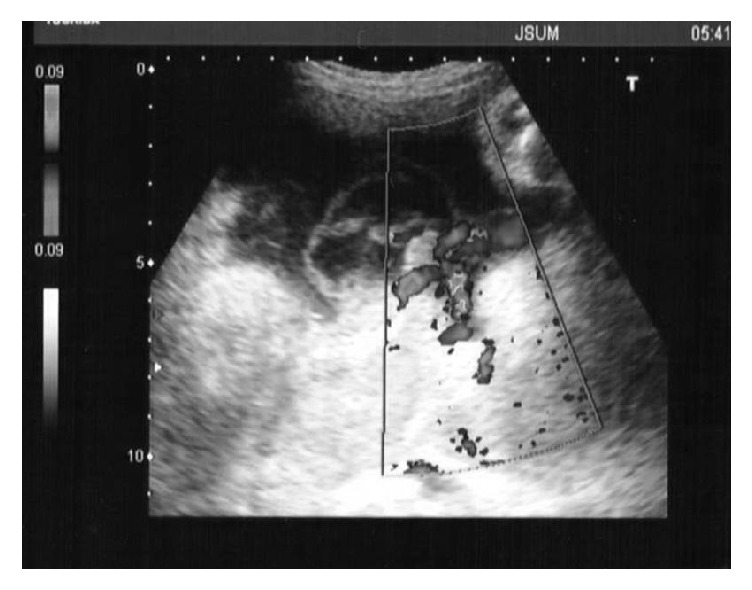
Ultrasonographic image showing subamniotic hematoma at 27 weeks in Case 4.

In 7 of the 9 cases (78%), the subamniotic hemorrhage was shown as oval-shaped mass wrapped in the amniotic membrane at the placental cord insertion site in the fetal surface of the placenta (Figure 1), while it was shown as marginal irregular traces of bleeding under the broken amniotic membrane in 2 cases (22%, Figure 2). In our institute, the screening of placental hemorrhage has been routinely performed using ultrasound in all cases. In the former cases, the subamniotic hemorrhage was shown as cystic lesion protruding from the fetal plate by ultrasonography during the pregnancy (Figure 3), while any abnormal findings were not recognized by ultrasonography in the latter cases. In the former cases, severe fetal growth restriction and preterm delivery were occurred in 2 (29%) and 4 cases (57%), respectively. In Doppler ultrasound examination, the decreased cerebroplacental ratio was observed in the 2 cases of fetal growth restriction; however, the middle cerebral artery-peak systolic velocities were normal. In addition, there were no abnormal findings of placental pathology leading to the development of fetal growth restriction except subamniotic hemorrhage.

On the other hand, there were no events until the onset labor pains at term in the latter cases. In Case 8, the neonatal hemoglobin concentration was 10.9 g/dL (normal: 13-22 g/dL) with reticulocyte counts of 0.9% (normal: < 7%). Although the amniotic fluid was bloody, any findings suggesting abruption or ulceration in the placenta were not recognized. The maternal hemoglobin-F was 0.5% (normal: < 1.0%). In Case 9, intrauterine fetal demise was recognized when the mother visited the hospital due to onset of labor pains at 38 weeks' gestation. The skin of the neonate was pale. Any findings suggesting abruption or ulceration in the placenta were not recognized and the maternal hemoglobin-F was normal (0.6%). Therefore, Cases 8 and 9 were clinically diagnosed as acute anemia associated with acute subamniotic hemorrhage. By placental pathology, there were no abnormal findings except subamniotic hematoma in the 2 cases.

On the other hand, the presence of gradual subamniotic hemorrhage in the second trimester of pregnancy was suspected in the former cases (Cases 1-7). In the 7 cases, there were no neonates complicated by anemia or adverse long-term outcomes.

## 3. Discussion

This may be the first report examining the clinical characteristics of acute or gradual intrauterine subamniotic hemorrhage.

The gradual subamniotic hematoma appeared as an oval-shaped soft mass wrapped in the membrane, while acute hemorrhage was recognized as marginal irregular traces of bleeding under the broken membrane. In the latter, the acute massive hemorrhage was thought to cause the collapse of the thin amniotic membrane, and then the bloody amniotic fluid was generated in Case 8. On the other hand, the difference in pathogenesis between acute and gradual subamniotic hemorrhages has not been well examined. Based on the difference in clinical courses, we speculate that acute subamniotic hemorrhage is related to the presence of uterine contraction leading to external pressure while gradual subamniotic hemorrhage occurs spontaneously by the intravascular pressure at a focus of lower vascular wall resistance. However, Van Den Bosch et al. [4] reported that the case of gradual subamniotic hematoma was suggested to occur due to the episode of premature contraction. Cases of intrauterine subamniotic hemorrhage are rare; however we expect the elucidation of pathogenesis of acute and gradual subamniotic hemorrhage by accumulation of the same case reports.

In some earlier literatures, subamniotic hemorrhage has not been thought to cause severe intrapartum complications [2, 4] except the cases of acute massive hemorrhage [5, 7]; however severe fetal growth restriction and preterm delivery occurred in 2 (29%) and 4 cases (57%), respectively, in our cases with gradual subchorionic hemorrhage. In this study, the fetal growth restriction might be due to the insufficient blood supply to the fetuses associated with the subamniotic hemorrhage based on the findings of the Doppler ultrasound examination. Classically, it had been thought that large hematoma of average cyst size was larger than 4.5 cm, more than 3 in number or at the placental cord insertion site leading to fetal growth restriction and nonreassuring fetal status [1, 8–10]. In recent years, however, the conception has tended to be denied [2, 4]. Unfortunately, we could not find any factors affecting the adverse perinatal outcomes associated with the presence of gradual subamniotic hematoma in the clinical courses or placenta findings of our cases. Therefore, a strict fetal surveillance including frequent Doppler ultrasound examination may be needed in all cases complicated by gradual subamniotic hematoma.

We present here the clinical characteristics of acute or gradual intrauterine subamniotic hemorrhage. However, we understand that the sample size of this study is too small to examine the etiology or pathology of intrauterine subamniotic hemorrhage as one of serious limitations because the sequence is very rare. To clarify the etiology and pathology of intrauterine subamniotic hemorrhage, a further study with accumulation of the same case reports is needed.

## Figures and Tables

**Table 1:** Cases of subamniotic hemorrhage managed at our hospital.

Maternal age (y)	Parity	GA at diagnosis (w)	Ultrasonographic findings	GA at delivery (w)	Delivery mode	Birth weight (g)	Macroscopic finding of the placenta	Apgar score (1/5 minutes)	UApH
34	0	19	Cystic lesion protruding from the fetal plate (4x3x2 cm)	38	Vaginal delivery	2,954	Oval-shaped mass wrapped in the membrane	8/9	7.254

40	2	21	Cystic lesion protruding from the fetal plate (4x3x3 cm)	38	Vaginal delivery	2,742	Oval-shaped mass wrapped in the membrane	9/9	7.198

36	1	22	Cystic lesion protruding from the fetal plate (4x4x3 cm)	40	Vaginal delivery	3,128	Oval-shaped mass wrapped in the membrane	8/9	7.289

26	0	24	Cystic lesion protruding from the fetal plate (6x4x3 cm)	33	CS due to nonreassuring fetal status	1,254 (SGA)	Oval-shaped mass wrapped in the membrane	4/7	7.157

24	0	27	Cystic lesion protruding from the fetal plate (5x4x3 cm)	27	CS due to labor	678 (SGA)	Oval-shaped mass wrapped in the membrane	5/8	7.200

27	0	27	Cystic lesion protruding from the fetal plate (6x5x3 cm)	34	Vaginal delivery	2,154	Oval-shaped mass wrapped in the membrane	8/8	7.169

24	0	29	Cystic lesion protruding from the fetal plate (4x4x3 cm)	30	Vaginal delivery	1,426	Oval-shaped mass wrapped in the membrane	6/8	7.118

31	1	37 (after delivery)	No abnormal findings	37	CS due to nonreassuring fetal status	2,618	Extensive subamniotic hemorrhage	2/6	7.012

33	0	38 (after delivery)	No abnormal findings	38	Still birth	2,599	Extensive subamniotic hemorrhage	-	-

GA, gestational age; CS, cesarean section; SGA, small for gestational age; UApH, umbilical artery pH.
